# Struggle over control: Sound in home video

**DOI:** 10.1177/13678779221135057

**Published:** 2022-11-07

**Authors:** Renée Winter

**Affiliations:** 27258 University of Vienna, Austria

**Keywords:** age, audiovisual practices in the home, conflicts in the private sphere, control over sound, gender, home video, sound

## Abstract

This article investigates sound practices in home video. Home video manuals and magazines recommended specific strategies for dealing with sound, often with the goal of gaining control over the openness and unpredictability of the situation being filmed. The subject of home video discourse (addressed in handbooks primarily as white, male, and the father of a family) was ideally the one that has image and sound well under control. But while manuals promised the possibility of (re)gaining control over home video, examples of recordings show the ultimate failure in realizing such a possibility. The article argues that listening to home videos can give insight on how media practices inscribe themselves into everyday life and are, therefore, linked to power relations, attempts to control, and scopes of action within the domestic sphere.

The 1980s made video cameras, a technology used primarily in professional or activist settings up to this point, available and affordable to a growing number of people. Unlike photography and small gauge film, the predominant visual media for documenting everyday and private life up to this point, the use of video also included synchronous sound as standard. This article aims to explore the sound of home video as a product of cultural practices that are shaped by historically specific technological, ideological and social conditions.

In the following, references to ‘home video’ mean an audiovisual practice in which video recordings on magnetic tape were made in the non-professional (and non-activist) sphere – especially during the period from the 1980s to the 2000s. Moran (2002: 36) clarifies his use of the term ‘home video’ as the ‘amateur practice of video in the home mode’, thus referring to Chalfen's (1987) study of snapshot photography in which he describes the ‘home mode’ as ‘a pattern of interpersonal and small group communication centered around the home’ (1987: 8). Chalfen (1987: 61f.) makes it clear, however, that many recordings in the home mode are not taken at home at all, but in nature, at religious places or in (semi-)public spaces, when filming during holidays, birthdays or other significant events.

Roger Odin (2014: 16f) describes two modes of reception of home movies that correspond to two forms of memory: private and intimate mode. In the shared private reception in the group, a common meaning is established by talking about the recordings together. In the silent and unspoken, however, the individuals in the group create their own narrative about the family history and their role in it, says Odin. Video sound promotes this intimate mode of reception because, unlike silent film, it does not invite the viewers to fill the silence with conversation, but rather requires them to listen to the sound. I would argue that for this form of intimate non-common-sense reception, listening to the unintended sounds of the home recordings can provide significant clues. Cubitt (1993: 122) speaks of ‘the contingent, the risky, the opening up of sound worlds’, which would be denied by a ‘wall of sound’ formed by a soundtrack of music and spoken words. Listening to the uncontrollable sounds that are considered as ‘noise’ and which result from synchronous sound recording, is also a promising perspective in a historical analysis of home videos that tries to read the documents against the grain of their intention.

After a brief overview of the history of family audiovisual practices in the home and private sound recordings, I discuss the sources and methods underlying my research. The section ‘Perceptions of sound in video manuals’ explores practices of dealing with sound in home video by asking how problems with sound (recording) were addressed in home video manuals in which synchronous sound was often referred to as something to be controlled. In the following section I analyse sounds of home video recordings from publicly archived home video collections in order to gain insight into home media practices. I understand the home, the domestic sphere, as a sphere historically shaped by power relations structured by gender and age (Morley 2000). In the last section, I make the case for the value of listening and tuning in to the uncontrollable sounds of home videos, not in seeking to get a more authentic historical picture of families, but to listen to hidden conflicts and struggles over control within the domestic sphere that are manifestations of (re-)negotiations of media practices and/in orders of the home.

## Historical constellations of family, gender, and audio/visual practices

In his study *Home Territories*, David Morley (2000) traced the historical development of the idea and implementation of the home as a private family space, which is closely linked to the popularization of a bourgeois patriarchal family model. As he shows, the construction of the home is characterized by many, often implicit, regulations and rules structuring the space (Morley, 2000: 18–21). The rules organizing the spatial structure also affect its sound, its auditory order. According to LaBelle (2010: 51), the home is associated with ‘auditory clarity, where order is equated with quiet, and the maintenance of domestic life with audible regulation. To come home is to seek refuge, however consciously, from the uncontrollable flows of noise and the harangue of the exterior.’ In LaBelle's account, the disturbing ‘other’ is primarily located outside the home; however, home video discourse locates the threat to auditory order that also comes from within. For instance, amateur film clubs (such as the *Klub der Kinoamateure Österreichs* founded in 1927), which initially showed a great deal of scepticism toward the video technology of the 1980s, problematized specific technological features such as long recording times and synchronous sound. In 1986, an amateur filmmaker described the problems of synchronous sound in an article about the advantages and disadvantages of video technology compared with film as follows:

The original sound, which is so fascinating at the beginning, certainly gets on your nerves at some point. That eternal car noise in the background, the squeaking firewood saw of the neighbor who just had to cut his wood during the beautiful scene at the badminton game, and the inappropriate remark of my wife, ‘Are you recording right now?’ when Gruber Jr was playing so videogenically in the paddling pool. (Gruber, 1986: 4f.)^1^

In the production of an audiovisual representation of family life, then, family members can function as a disturbance. What we see here is a gendered ordering of the film subject, the filmed object, and the disturbance, an issue to which I shall return.

Concepts of privacy, family, and home are inscribed and negotiated in selling strategies, advertisements, and discourses about the popularization of media technology (Aasman, 2009; Zimmermann, 1995). As Susan Sontag (2004: 6) observed about photography in a family context:

[p]hotography becomes a rite of family life just when, in the industrializing countries of Europe and America, the very institution of the family starts undergoing radical surgery. As that claustrophobic unit, the nuclear family, was being carved out of a much larger family aggregate, photography came along to memorialize, to restate symbolically, the imperiled continuity and vanishing extendedness of family life. Those ghostly traces, photographs, supply the token presence of the dispersed relatives. A family's photograph album is generally about the extended family – and, often, is all that remains of it. (6)

This thesis of a correlation between the ‘vanishing extendedness of family life’ and its visual enactment and reformulation is significant for this study in two ways. First, it points to the fact that ‘the family’ is not simply the object of the visual record; audiovisual practices interact with, are shaped by, and shape constructions of familiality. Second, it necessitates that any analysis of videos in familial contexts must relate them to social changes, changes in family structures, and changes in the constructions of ‘family’ since the late 1970s. The linkages of media technologies with representations of the family and everyday life have a long history. The Lumière brothers’ film *Le Repas de bébé*, for example, was part of the first ever public screening of films in 1895 and according to Melinda Blos-Jáni (2015: 112), can be used to show how ‘the discourses of realism of early cinema were linked to the discourses of immediacy of home movies’. The constellations of media, gender and family constructions are subject to historical change and thus are to be examined specifically in each case.

The spread of video technology took place in parallel with and through interaction with social changes that affected ideas of private life, of living together, and of family and affiliation. Aasman (2012: 166) asks ‘to what extent [do] new media technologies uphold family life that is no longer localized in a space or particular time?’ Video as used in the home mode is thus also intrinsically connected to changing concepts of family, gender, and sexuality. As commercials for consumer electronics show, home media practices can work to stabilize traditional structures of reproduction work and wage labour by promising the ability to make up for missed moments – to repeat and relive them. However, home media technologies can also be used to destabilize and challenge gender relations. Zimmermann (1995: 39) has shown, for example, that in amateur photographic discourse around 1900, women were seen as ‘superlative image-makers’ because they had the ‘patience and time to delve into artistic, pictorial photographs’. This attribution alleged women to be ideal photographers of the home and daily life. For some women, however, this domestic engagement with photography offered the possibility of a transition to professional photography, as Zimmermann argues (1995: 39f.). The ‘Kodak girl’ in particular, who had been in widespread use as an advertising icon for Kodak's small cameras since the end of the 19th century, achieved great public visibility. As Tim van der Heijden (2018: 137–42) has pointed out, the establishment and popularization of Super-8 film technology also largely addressed women as new users, who were to be appealed to in particular by the easy manageability of the small cameras. However, as van der Heijden argues, these representations of women as users are accompanied by a paternalistic discourse and ‘women were usually represented as first-time users interested mainly in the recording of family memories, whereas men were often portrayed as serious hobbyists’ (2018: 40).

Discourse surrounding representations of everyday life in home videos was shaped by white, middle-class ideals of family, as an analysis of manuals and journals reveals (an idea to which we shall return). Notions of what events and motifs are worth recording were also based on historical predecessor media, such as family photography and small gauge film. In his publication on family cinema in Germany, Kuball (1980: 119) contrasted the bourgeois film amateur, who in the 1920s and 1930s was preoccupied with filming the home, vacations, and the car, with the proletarian film amateur, who made films about political events, worked collectively, and organized. Ladwig (2018) commented on the Austrian amateur film practice of the interwar period, writing that ‘the performative act of family film practice (re)produces […] family – and, in a sense, reflects the privatized and emotionalized concept of family as a supporting bourgeois model in society’ (2018: 84). Odin (2014: 16) described home movie-making in the postwar period as being rooted in the bourgeois patriarchal family:

Within this structure, the father has a particular position; it is he who directs the formation of familial memory; it is he who oversees the building of the cemetery grave; he who orders the painted family portraits; who takes the photographs; and, obviously, it is he who shoots the films.

In his seminal work on home video, Moran (2002) pointed out that although familialist ideology was deeply inscribed in the practices of home movie-making, the same was not so true for home video. For one, the ideology of the nuclear family was increasingly challenged in the 1980s and 1990s; for another, the specifics of the medium of the video were also different (2002: 35–9). He argues that lengthy recording times in video and the fact that ‘the camcorder fits more easily into everyday life without intervening in routines, selecting content, or posing subjects’ enabled the presentation of a wider range of domestic life, compared with small gauge film (Moran 2002: 42). ‘In short,’ Moran notes, ‘home video reveals that families have always been more complex and contradictory than home movies have generally portrayed them’ (2002: 43). Similarly, Odin (2018: 26) argues that ‘[t]he home video features family life *as it is*, including its happy moments, but also all its pettiness, all those moments of rivalry and conflict that will always occur in any group’. Owing to synchronized sound, in particular, home videos contained scenes that would have been ‘unthinkable’ in home movies, ‘such as words one would have preferred to forget, unpleasant remarks, and arguments’ (Odin, 2018: 26).

Media theorists have shown (e.g. Blom, 2016; Moran, 2002; Spielmann, 2005) how the distinct properties of video clearly distinguish it from (small gauge) film. Characteristics such as immediacy, instant playback, eraseability, replayability, affordability, and synchronicity form the basis of specific home video practices. These practices include extended recording times, looking at oneself on the monitor while recording, making recordings that could be deleted later but then keeping them anyway because the material is so cheap, turning on the camera unnoticed by others, and looking at the result immediately after recording.

Moving beyond claims of greater realness and authenticity in home video thanks to video technology, I want to shift our focus towards the productive and transformative functions of synchronous sound in home video, and the role video technology plays in private-sphere power relations. The following section considers how, and to what effect, sound recordings have entered the home.

## Private sound recordings

In the 1980s and 1990s, sound recordings in the private sphere were not an entirely new phenomenon. Already Thomas Edison included family recordings, ‘a registry of sayings, reminiscences, etc., by members of a family in their own voices, and the last words of dying persons’, in a list of possible uses of the phonograph, as Jonathan Sterne (2003: 202f.) has noted. From the late 1940s, tape recorders had been produced for non-professional use and thus were also used in private and family contexts. In a study of the domestication of tape recorders, Bijsterveld and Jacobs (2009) noted that when tape recorders first entered households in the 1950s, they were advertised as a tool for creating acoustic family albums. While users increasingly used tape recorders to record and play back music (from the radio), manuals and advertisements suggested recording children at play, songs, or snoring grandfathers (Bijsterveld and Jacobs, 2009: 25f.). Compared with silent amateur photography, the sound was intended to make (re)experience possible; advertisements emphasized how the recorded sound was both immediately available and playable, as well as erasable at any time (Bijsterveld and Jacobs, 2009: 29).

The characteristics of immediate availability alongside the eraseability of recordings were something that video recording shared with the audio tape. The magnetic tape upon which video recording is based was originally developed and used for recording sound, as pointed out by Filiciak (2018: 138f.; see also Armes 1988: 110–12). Through video technology, synchronous sound recording became normal. Although the first amateur sound film systems had been created in the 1920s and 1930, synchronous sound and image recording became widely accessible for private use only with the medium of video (van der Heijden, 2018: 156). This is because synchronous sound on small gauge film had turned out to be less practicable. For instance, a 1983 manual comparing Super-8 and video explains that

[w]ith direct-tone S-8 film, it is no longer possible to work properly in a truly cinematic way. For direct sound recordings, the long recording spans provided by the video cassette are absolutely essential. […] Direct sound recording with Super-8 had become technically simple, but was almost impossible to master creatively. This was precisely because one couldn’t hold the individual shots long enough. (Spitzing, 1983: 34)

Van der Heijden (2018: 203) notes that synchronous sound recording brought new problems: not only did the video camera itself produce unwanted sounds that were audible on the recording, but instructions from the camera person were also made more difficult. Given the length of the recording time, sound recording also lent a feeling of being under surveillance to those being filmed in private settings. People dealt with the new demands and affordances of synchronous sound by modifying already established practices and developing new audiovisual ones.

Having established the links between audiovisual media, especially sound recordings and the private sphere, I will now present the sources and methods forming the basis of my analysis.

## Sources and methods

My analysis is based on two bodies of source material: home video manuals and home video recordings. The examination of home video manuals draws on 19 handbooks published between 1980 and 1993. The majority of these were produced for the German-speaking world (Bänninger, 1987; Förster, 1982; Folgner and Birke, 1992; Frese, 1982; Freymuth, 1989; Glogger, 1983; Gruber and Vedder, 1982; Kämmer, 1991, 1993; Maschke and Bülow, 1991; Möller and Ullrich, 1991; Schild and Pehle, 1992; Spitzing, 1983; Von Lichem, 1983; Wild and Möller, 1990), and one was translated from Dutch (Wezel, 1980). Three manuals were originally written in English and translated into German, Italian, French, and Spanish (Owen and Dunton, 1983), Dutch, Italian, Finnish, and German (Dollin, 1986), and Italian, Spanish, Finnish, German, and Chinese (Hedgecoe, 1992). The translation practices of manuals indicate the transnationality of home video discourse as well as the centres and directions of travelling concepts within this discourse. Most of the manuals assume little or no prior knowledge and are thus aimed both at inexperienced users and, in later chapters, at amateurs who have already worked with video. Four of the manuals (Folgner and Birke, 1992; Freymuth, 1989; Kämmer, 1993; Wezel, 1980) are aimed at the experienced videographer. The handbooks were selected on the basis of their presence in university libraries and online antiquarian bookshops in a German-speaking area; they are thus indicative of published and available home video knowledge.

I also analyse a body of home video recordings. Home video recordings are rarely represented in audiovisual archives. The digitization of a variety of now-obsolete video cassette formats requires a great number of recorders and players, in addition to very specialized knowledge, to be able to treat the many different formats and signals. Most video collections in archives are limited to professionally recorded videos, such as artist videos, broadcast materials, or television recordings. The Österreichische Mediathek (OEM) and Filmbank Groningen have both archived a considerable number of home videos, however. As Austria's national audiovisual archive, the former holds a collection of more than 3000 video cassettes – mainly amateur, home, and private videos from about 100 different videographers in eleven different formats (VHS, VHS-C, S-VHS, Mini-DV, Hi-8, Video-8, Digital-8, Betacam SP, Betamax, Video 2000, Umatic) from the 1980s, 1990s, and 2000s. The files have been catalogued and keywords, date, location, and persons appearing in the videos are recorded in the catalogue. In some cases, the contact information of video donors can be used to obtain more detailed information about the video material as well as to arrange interviews. From this inventory, I screened roughly 1000 videos. Of these, I selected 300 from 23 different sources for closer analysis. I conducted narrative interviews with two videographers (who had digitized 51 and 28 video cassettes, respectively) about their home video practices. I also included videos from the Filmbank Groningen (formerly known as the Gronings AudioVisual Archive or GAVA). During an archival research stay in Groningen and with support from local archivists, I selected 20 home and amateur videos that complement and contrast the Austrian volume of work. Selection criteria for the videos mainly focused on media practices: the material should allow questions to be answered about video-specific audiovisual practices, their subjectifying functions, about how they are inscribed into everyday life – and thus how they are further linked to power relations in the home as a gendered sphere.

Analysing manuals and video enables us to grasp the normative discourse surrounding home video, as well as to understand actual media practices of everyday life. The following section focuses on how handbooks address sound.

## Perceptions of sound in video manuals

Although sound recording was closely linked to the development and use of video technology, video – in keeping with its name – was addressed in manuals and advertisements (as well as in many of its uses) primarily as a visual medium. Sound was a ‘neglected media dimension of meaning-making,’ according to Traue (2013: 284). In manuals, sound was usually assigned its own chapter that addressed sound recording along with dubbing. These were dominated by descriptions of glitches, unwanted sounds, and recommended strategies for gaining control over recorded sound (e.g. using external microphones). For the home videographer, problems lurked everywhere:

shooting indoors gives you most control over extraneous noise […] but the room can give trouble. […] Outdoor recording does away with worries about acoustics, but you can be plagued by all manner of unwanted noise from hammer drills to passing aircraft. (Owen and Dunton, 1983: 164)

The most prominent source of noise disturbance in home video is wind, which is problematized in almost all of the manuals. What is also disturbing is the buzzing and humming of the refrigerator, hot water heater, and air conditioner – all sounds of a mechanization of everyday life that is, at the same time, the basis of home video practice. The sounds of the camera itself are also consistently described as a disturbance. Accordingly, the technology that makes the recording possible should not be noticeable or even audible. As Birtwistle (2010: 86) notes about technical discourse on film sound, the sound of technology was designated as ‘system noise’ and framed as ‘problem to be solved, a sound that needs to be minimized even if it can never be removed entirely’.

‘Intrusive’ or ‘extraneous’ noises (Hedgecoe, 1992: 28), such as telephones, doorbells, traffic, airplane engines, construction sites, sawmills, hammering, and door slamming, were also identified as sounds that should be avoided. However, contradictions become visible in themes attributed to the context for shooting. Car rides, for example, represent a welcome and frequent motif – but associated engine noise should be avoided as much as possible (Schild and Pehle, 1992: 42). Still, the dividing lines between desirable and undesirable noises are flexible; as a manual titled ‘The Videomakers’ notes, ‘what one person perceives as euphony is noise for another’ (Bänninger, 1987: 103). This emphasis on the position of the listening subject is also echoed in sound research. Historical research on ‘noise’ shows that the perception of noise changed with industrialization. Which sounds are perceived as noise varies over time, across cultures, and among individuals. Noise is thus ‘a social category’, according to historian David Morat (2013: 138).

The manuals express two additional demands on sound recordings. First, the home videographer should avoid a ‘jumble of sound’ (Schild and Pehle, 1992: 148) and establish congruence between image and sound. In other words, what is seen should also be heard and vice versa (Maschke and Bülow, 1991: 148; Wild and Möller, 1990: 88). According to the manuals, the unambiguous connection of source and sound – which is questioned in experimental film and theory (Birtwistle, 2010: 42–3) – must absolutely be established. Thus, one function of recording is to create order and avoid ambiguity. As one manual prescribes, ‘always try to ensure that the sounds your audience hears are in character with what they see’ (Owen and Dunton, 1983: 114). Sounds judged disturbing in this sense are ‘[f]oreign/strange [*fremde*] noises that do not fit the picture’ (Bänninger, 1987: 50), which ‘have nothing to do with the actual scene’ (Förster, 1982: 118). The congruence of image and sound ultimately serves to establish authenticity; the manuals largely agree that original sound is more authentic (see Bänninger, 1987: 150; Freymuth, 1989: 147).

Video recording with sound has been the subject of broad discussions around possibilities for design and influence. Making recordings appear ‘natural’ is a goal – one that can be achieved with technical aids, precise advance planning, and physical practices, or, as Roy Armes (1988: 168) put it ‘the quest for “natural” sound involves a total manipulation’. Here, authenticity is the result of a differentiated production process. Much of this process is concerned with excluding everyday noises that are perceived as disturbing. This also gives an idea of how much work goes into creating ‘authentic’ family representations. At the same time, video manuals perceive only those noises that cannot be brought under control as undesirable. When purposefully used in dubbing, the same noises (that of traffic, doors, airplane) may in fact be perceived as desirable. Here the perspective on un/desirable sound is quite similar to what Levy and Pinchevski (2017: 3362) described in analysing sound in television news, where control over sound is a ‘a sign of journalistic professionalism’ and an expression of the purpose ‘to maintain authority’. In home video discourse the struggle over sound is part of a field of negotiation over authority and power relations within the domestic sphere. The subject of home video practice in the manuals is ideally one who has image and sound under control. The point is to ‘get a handle on the sound’ (Maschke and Bülow, 1991: 143) in order to obtain ‘control over unwanted noises’ (Dollin, 1986: 72), eliminate interference, and learn about ‘hazards you may not be aware of – like the saw mill next to the church’ (Dollin, 1986: 77).

But just who is this subject exercising control over the recording situation? In the majority of the video manuals under study, the filming subject is addressed as male and white, a father and a husband. On the one hand, this occurs on a visual level (as in John Hedgecoe's *Complete Guide to Video*; Hedgecoe, 1992: 20–3; see also Maschke and Bülow, 1991; Von Lichem, 1983).^2^ On the other, this representation is encoded within the text itself. Förster (1982: 75), for instance, addresses his readers thus:

Look through the viewfinder of your video camera and discover everyday things to which you hardly paid attention before. Look at the happy faces of your children, the new hairstyle of your wife. All you have to do is press a button, and your family's life is captured on videotape.

Similarly, Glogger (1983: 143) promises that ‘whether you’re on vacation, at parties, playing sports or recording music groups, you’ll always be welcomed everywhere as the man with the camera. There are few people who don’t want to see themselves on screen.’ Likewise, a manual titled ‘Family and Children in Videography’ identifies other subjects operating the camera as alternatives only:

The camcorder is the tool of the trade here. Often it will be the father who is intensively involved with the technology and therefore usually operates the camcorder. That should be left to him. But he should remember that such a camcorder aims at the simplest operation and, after a few explanatory words, can be used by every member of the family – by the grandmother as well as by the grandchild. And they would also like to film [‘*Und die möchten auch einmal filmen*’]. This takes the pressure off the father and gives him the opportunity to act as a performer himself for once. (Maschke and Bülow, 1991: 9)

Although the camera is passed from one subject to another here, ‘the father’ remains the primary owner and administrator of access, as well as the intra-family expert in its operation. Giving the camera away extends the possibility of the father entering the frame, a goal for which some manuals recommend using a tripod instead.

Stable patriarchal relationships further prevail in video manuals with regard to suggested video motifs. Home video practice itself emerged in the 1980s and 1990s, which was a time when traditional familial structures and gender representations became more persistently contested (not least owing to the women's movements of the 1970s and LGBTIQ activists). But, surprisingly, the motifs proposed in handbooks on home video differ little from family representations in earlier, small-gauge film practice. For example, the 1991 handbook ‘Family and Children in Video Films’ suggests the following:

To give you a rough idea of the kind of events you shouldn’t miss in the next few years if you have children coming into the house now: birth; the first steps; the first and subsequent birthdays of children; playing together on the playground, at home, and with friends; the arrival of Santa Claus; the first Christmas; Easter egg hunts; the first encounter with an animal; the first day at kindergarten; the first day of school; communion/confirmation; high school or apprenticeship; graduation from high school or passing an exam; the first apartment; the wedding; and the arrival of grandchildren. (Maschke and Bülow, 1991: 105)

Here, the ideal life course of a white, Christian, heterosexual subject is drawn – a subject that can be integrated into the institution of education and the labour market. Life in home video appears cyclical. It follows its predetermined course with predictable (annual) events and milestones. At the end of the home video life recorded by the father, new children appear. This marks the beginning of a new video cycle.

## Sound practices in home video recordings: Archival findings

The normative discourse of the manuals transfers into home video practices only to a limited extent. Video collections in audiovisual archives show how the people actually doing the filming were much more diverse than those addressed in the manuals. Video cameras were regularly passed from person to person and the actual historical audiovisual practices were more diverse in terms of the subjects of film, the choice of motifs, and cinematic techniques.

The videos reveal several tactics for bringing sound under control. Ambitious video makers dubbed their videos with either music or spoken explanations and stories. On-camera narration is the most commonly used practice to not only control sound, but also narrate the video. As Slootweg (2018: 214) points out, this particular option was also open to other persons present in the room, who could add verbal remarks or sounds because of the omnidirectional microphone, thus contributing to the narration of the scene.

The home videos also show that video was primarily used as a visual medium. What is in the picture seems to be more important to the subject of the film than what one can hear. This preference can bring about unexpected effects. One videographer filmed a tank truck filling up at a gas station in front of his house with audio of radio news about the 1987 Austrian Formula 1 Grand Prix and a weather forecast playing in the background.^3^ In another video, there are ducks on a pond filmed through a window accompanied by the music of television or radio inside the room.^4^ Friends gossip tipsily or merrily about relatives over the course of a barbecue.^5^ Children swear angrily at the parents filming them.^6^ Pejorative remarks and cruel words are hissed between spouses.^7^ Such sounds often tell a story that differs from the one related by the visual recording. They tell about conflicts and other elements of events that did not catch the eye of the videographer – about political realities when the television or radio is switched on, about kids present in the room, and about climate conditions. Another set of audible remarks relates to the context for filming, wherein the viewer constantly hears instructions either from (e.g. to do or not do something, to say something, to wave, to smile) or to (e.g. to film this or that, to stop filming) the videographer. The people on film often point out to each other that the camera is running,^8^ for instance, or someone may say something with the intention of it being recorded.^9^

Although home video is predominantly used as a visual medium, an analysis of archival material furthermore reveals its specific use as an auditory medium. Sound recordings are not limited to obvious uses, such as the recording of songs or theatrical events. In the home, the camcorder is also used to record evidence of mundane events, such as persistent snoring^10^ or promises from teenagers to do well at school.^11^ Sound is also important to videographers trying to capture the pronouncement of marriage at a wedding ceremony, a speech at university graduation ceremony,^12^ or a poem recited at a birthday party.^13^ In an interview about her practice, home videographer Brigitte S. recounted the problems she faced when attempting to bring sound under control. When filming birthdays, for example, she would try to situate herself in a position that would allow for a good visual overview. However, good visual positions were often too far away from speakers for the viewer to understand what they were saying or to avoid the noises and murmurs of party guests^14^ (like most of the home videographers who donated videos to the Österreichische Mediathek or the Filmbank Groningen, Brigitte S. didn’t use an external microphone). She also showed concern over her own vocal expression captured on film:

And most of all, uh, with the films, it is so that … I do fall into a dialect at times. And I like to be quiet in the background, because then you hear everything, such as, uh, whether you speak beautiful high or standard German, and things like that. That is then, uh, already a bit of stressful. I control myself with language, and so on …^15^

Here, control over sound becomes self-control over class origins that are unintentionally revealed in dialect or the use of slang. The videographer perceives these origins as ones that should not be heard on video. Although the home video is not being produced for the public at large, the auditory self-construction is a desire to speak ‘beautifully’. Much like visual motifs oriented to bourgeois ideals, the video is also meant to sound beautiful. Buckingham et al. (2011: 93f.) further mention an alienating effect caused by the perception of the representation of oneself on video. For some, this is produced in the process of hearing themselves (as opposed to seeing themselves) on screen. In response to an interview question about listening to her own voice, Brigitte S. was self-observant and evaluative of how she spoke, concluding that she was generally satisfied with the sound of her voice.^16^ For this historical period, too, the visual representation of the self in home video was nothing new: people were long familiar with their own image by way of mirrors, photographs, and (less commonly) small-gauge film. However, audio recordings of their own voices were not as common.

Last but not least, there are sounds recorded without intention of the videographer. The inadvertent murmur of the camera being switched on, voices, the sound of footsteps while filming the floor, the wall, or the inside of the camera cover are all captured on home video.

## Listening to noise

Media scholar Pooja Rangan (2017) uses the term ‘audibility’ in reference to French philosopher Gilles Deleuze's remarks on ‘visibility’. For Deleuze, visibility is not a quality inherent to objects; rather, it is produced by the light that makes the object visible. Writing about voices in documentary film, Rangan (2017: 282) argues that the voice can be similarly understood as a product of specific listening practices that give meaning to sound. So, what can we hear when we listen to home videos?

In the home videos under study, the fantasy of control over sound rarely plays out. What becomes audible instead are many unplanned disruptive sounds and voices. While what the viewer sees (if not the behaviour of the subjects before the camera) can be largely brought under control by framing, this tactic does not succeed when it comes to sound. There is talking during filming, ranting, singing, and matters being discussed that should not be recorded. The television and radio are on. Instructions are given from the camera person to the others and vice versa. As shown earlier, the cinematic practices of people behind the camera are often directed at the image (unless the image is explicitly about the sound, such as with a concert, a child's poem, or a speech).

One could say that there is a great deal of noise in home video. As already mentioned, Birtwistle (2010) observed that the system or ‘ground noise’ of film camera and projector has been framed as a problem; he further notes how ‘the assumption is that the sound of technology is something that is listened *through* rather than listened *to*’ (2010: 88, italics in original). In home video discourse, too, the sound produced either by humans or the environment is framed as noise. At best, this noise is absent; if it is present, we are otherwise supposed to ‘listen through’ it. But what happens if we don’t ‘listen *through*’ this kind of noise? What happens if we listen *to* it?

In his post-foundationalist social theory, Oliver Marchart (2013) proposes a particular reading of French historian and philosopher Michel Foucault's thematizations of social struggles. In this reading, Marchart (2013) describes these struggles primarily as acoustic phenomena. There are: ‘[m]urmurs, noises, rumblings, thunderings, shouts: the expressions of struggle in Foucault’ (2013: 436). Here, Marchart is explicitly referring to Foucault's theory of micro-levels of conflict. These are conflicts in everyday life – gendered conflicts in the domestic sphere and disputes between parents and children – the many clashes and interactions between domination and rebellion as expressions of social relations understood as political and power relations structured by conflict (Marchart, 2013: 433).

In order to understand the importance of listening to the unintentional – noise itself – I would like to describe two video scenes from the inventory of the Viennese family of Brigitte S. in more detail. The first scene is from a family vacation at the Wörthersee. During an excursion to the Pyramidenkogel, Brigitte S. pans across a panorama while describing the scene from off camera, saying, ‘From the Pyramidenkogel: Klagenfurt, Keutschacher Lake, Velden, the Wörthersee, Pörtschach, Maria Wörth, Klagenfurt, and Gerhard. Wave! Say hello!’ Her husband Gerhard S. grins at the camera and squeezes out a dismissive ‘goodbye! [*Auf Wiedersehen*]’ instead of returning a ‘hello’.^17^ While the picture is supposed to show a family outing set against beautiful landscape, the sound of their interaction reveals that the situation was anything but harmonious. In his study of the uses of amateur video, Slootweg (2018) encountered a similar scene. When analysing the home videos of a Dutch expat family, he describes how the sound level interferes with the father's attempt to create an audiovisual construction of domestic happiness (2018: 240–6). As his daughter inscribes her disagreement with the decisions of her parents via synchronous sound into the videos, Slootweg (2018) analyses the father's practice as ‘a product of “masculine domesticity” in which the “family” and “home” were communicated, captured and mediated by performing fatherhood’ (2018: 246) specifically ‘to counter the potentially harmful effects of continuous temporary migration on the stability of family life’ (2018: 246). Here it is the mother who wants to record a scene of a pleasant family holiday, but the husband does not integrate into the panorama as harmoniously as the mountains and lakes. Following Slootweg's analysis of the filming father, we can say that Brigitte S. uses the video camera to perform family life as it should be. While the performance is largely successful visually, it is interrupted on the sound level by the husband. The audibility of conflicts or differences can be found in many videos. Often, the camera is turned off when such expressions occur but, sometimes, it remains stubbornly on.

The second scene is one in which the threat and then enactment of physical violence against children is made apparent through sound. This time, husband and father Gerhard S. is filming. The setting is the garden of the grandparents during the summer of 1996. There, Gerhard's 7-year-old son and his nephew are running around in an agitated manner. His child runs to the camera, saying, ‘Don’t take a picture or I’ll flatten you!’ The child is then threatened with a slap by his grandmother, who doesn’t want to be filmed – as she states in the direction of the camera. A little later, the child jumps up and down in front of the camera. Standing behind the camera, the annoyed father says, ‘Stop it!’ The child approaches closer and is thus, for two seconds, outside the frame. At that moment, a snapping sound can be heard. The child screams ‘ow!’ and insults the father. The father replies, ‘That's all on film now.’^18^ Here, sound verbalizes non-consent with the recording; the slap, which according to the grandmother should not be part of the video, is included at the close of the recording. The discrepancy between normative visibility and audibility is particularly clear: a slap would not be an adequate motif for a family album. However, the video camera remains on throughout the scene. In fact, it is not even put down.

These two examples show the audibility of conflict as unwanted or unintended sound. At the same time, they also point to the intervening role of the camera within these scenes of conflict. What was humorously thematized in the ‘Dictionary of Video Filming’ manual (Möller and Ullrich, 1991) also points to a function of the camera. Under the keyword ‘director,’ the manual identifies the ‘[f]amily father who brings his relatives to acting excellence in the interest of a gripping film scene. With the help of a camera, fathers can playfully regain their already fragile authority’ (Möller and Ullrich, 1991: 62). The camera is used not only to produce certain images and visibilities, but also to intervene in situations. Thus, traces of conflict can be heard – but the subjects acting in front of the camera are aware of the normative demands of good visuality, which influence their behaviour. In this sense, the camera can have an appeasing function: it can be seen as an attempt to gain control over a situation. It can also offer limited protection from slaps in the face and evoke certain other actions, such as playing the flute at Christmas or hugging parents. It gives the videographer additional options for action in routinized, everyday, and celebratory situations. The director does not have to help out or participate in the conversation but is still able to be involved or have something to do.

Moreover, the loss of control over sound lamented in the manuals refers to the person behind the camera. For those in front of the camera, the synchronicity of sound opens up perspectives for action. They are co-producers of the audio representations of the family. Children, especially, exploit this possibility vigorously. The image selection of the videographer is further commented upon by those present (e.g. ‘You’ve only begun filming now, when you can’t see everything there was to eat any more’) who may also give instructions to the cameraperson (e.g. ‘Please go. Stop filming and help out now’).

## Conclusion

Home video on magnetic tape is now a historical audiovisual practice. Smartphones have largely replaced camcorders as filming devices and made practices of audiovisual recording an inseparable part of everyday life. Filming with smartphones is subject to conditions and requirements to a certain extent different to those of video cameras. Filming with mobile phones is often intended for immediate sharing via messenger or posting on social media, rather than storing it or creating a postprocessed audiovisual memory. Manuals in the form of handbooks have become rare but are often videos themselves. Filming the family no longer seems to be mainly the business of the father of the family. The 1980s to 2000s represent a transitional phase in which media practices of the home were renegotiated in interaction with the social orders of the private sphere.

As I have shown, the normative discourse of video manuals was chiefly concerned with developing strategies to control sound. Control over domestic sound was closely linked to patriarchal (understood as referring to both, to gender and to generation) notions of the home. Home videos not only show how these attempts regularly failed, but also highlight that, among these uncontrollable sounds of everyday family life is evidence of the conflicts that are hidden or undesirable in visual representations of the family. Where the cameraperson lost control over the sound in the home video, the other actors gained power and influence over the presentation. Listening to the sound of home videos brings us no closer to a supposed authenticity – to a historical truth about family life or more real representations. But it can help us understand how video became part of a field of conflict, how it has been inscribed with the power relations of the domestic sphere, and how actors endowed with different levels of power within this field made use of it.

## Notes

1. All translations of quotations are by the author.

2. While in the majority of manuals the whiteness of the filming subjects is suggested by their visualization, this presupposition is implicit in Hedgecoe (1992: 143), which addresses exposing non-white faces as a problem at the point when it comes to vacation shots (using the example of a dance scene in Gambia).

3. OEM: E07-01563.

4. GAVA: AV13367-2560-4815.

5. OEM: E07-00909.

6. OEM: E07-00905, E07-00658.

7. OEM: E07-00657, E07-00920.

8. OEM: VX-06882.

9. OEM: E07-00495.

10. OEM: E07-00905.

11. OEM: E07-00915.

12. OEM: E07-01242.

13. OEM: V-19351.

14. Personal interview Brigitte S., 26 February 2020.

15. Personal interview Brigitte S., 26 February 2020.

16. Personal interview Brigitte S., 26 February 2020.

17. OEM: E07-00915; 00:00–05:21.

18. OEM: E07-00658; 07:50–08:50.
